# Genomic characterisation of *Salmonella enterica* serovar Wangata isolates obtained from different sources reveals low genomic diversity

**DOI:** 10.1371/journal.pone.0229697

**Published:** 2020-02-28

**Authors:** Kelly M. J. Simpson, Siobhan M. Mor, Michael P. Ward, Julie Collins, James Flint, Grant A. Hill-Cawthorne, Moataz Abd El Ghany

**Affiliations:** 1 School of Veterinary Science, Faculty of Science, University of Sydney, Camden and Camperdown, Sydney, New South Wales, Australia; 2 Westmead Institute for Medical Research, Marie Bashir Institute for Infectious Diseases and Biosecurity, The University of Sydney, Westmead, New South Wales, Australia; 3 Institute for Infection and Global Health, Faculty of Health and Life Sciences, University of Liverpool, Liverpool, Merseyside, United Kingdom; 4 Hunter New England Population Health, Wallsend, New South Wales, Australia; 5 National Centre for Epidemiology and Population Health, Australian National University, Canberra, ACT, Australia; 6 School of Public Health, Sydney Medical School, The University of Sydney, Sydney, New South Wales, Australia; 7 Westmead Institute for Medical Research, The University of Sydney, Sydney, New South Wales, Australia; Defense Threat Reduction Agency, UNITED STATES

## Abstract

*Salmonella enterica* serovar Wangata is an important pathogen in New South Wales (NSW), Australia. The incidence of *S*. Wangata is increasing and transmission is suspected to be via a non-food source. A recent outbreak investigation of sources of *S*. Wangata recovered isolates from humans, domestic animals, wildlife and the environment. Here, we extend that investigation by characterising and describing the genomic determinates of these isolates. We found that Australian *S*. Wangata isolates from different sources exhibited similar virulence and antimicrobial resistance gene profiles. There were no major genomic differences between isolates obtained from different geographical regions within Australia or from different host species. In addition, we found evidence (low number of SNPs and identical virulence gene profiles) suggestive of an international transmission event between Australia and the United Kingdom. This study supports the hypothesis that *S*. Wangata is shared between different hosts in NSW, Australia and provides strong justification for the continued use of genomic surveillance of *Salmonella*.

## Introduction

*Salmonella enterica* is a Gram-negative bacterium associated with a wide range of disease outcomes in humans, domestic animals and wildlife. *Salmonella* infections vary from asymptomatic colonisation to gastroenteritis or severe, systemic disease [1, 2]. The disease outcome is determined by the pathogenicity and host specificity of the *Salmonella* serovar, as well as factors such as age and immune status of the infected host [2]. As with other enteric pathogens, *Salmonella* is also capable of surviving in a range of different non-host environments [3, 4].

*Salmonella* is a major cause of gastroenteritis in the Australian state of New South Wales (NSW). In 2018, the incidence was 42.6 notified cases per 100,000 people [5]. The most commonly isolated serovar is *S*. Typhimurium, which is responsible for almost half the salmonellosis cases notified in NSW [6]. Salmonellosis is predominately transmitted via a food source [6], however non-foodborne exposures, such as contact with animals, environment and water, are also important sources of *Salmonella* infections in humans [7, 8].

*Salmonella enterica* serovar Wangata is an important emerging serovar in NSW. The incidence of *S*. Wangata has been increasing [8] and in 2016 it was the sixth most frequently isolated serovar in humans in NSW [5]. Preliminary investigations have been unable to identify a common food source and it is hypothesised that this serovar is primarily transmitted via an environmental route [8].

In order to identify possible environmental and zoonotic pathways for infections of *S*. Wangata an investigation into this slow moving outbreak was conducted between November 2016 to April 2017 in north-eastern NSW [9]. The outbreak investigation had three components, with corresponding objectives: a case-control study to identify human risk factors for infection; animal and environmental sampling to determine if *S*. Wangata was present in the environment; and a phylogenomic analysis to explore the relatedness of human, animal and environmental isolates. There were 76 human cases identified during the outbreak period and 4 instances in which *S*. Wangata was isolated from the cases’ household environment. An additional 4 *S*. Wangata isolates were obtained from wildlife scats collected in parallel to the outbreak investigation. The epidemiological findings from this investigation have been reported in detail elsewhere [9]. These showed that indirect contact with bats/flying foxes, wild frogs and wild birds were statistically associated with human illness. However, the genomic characteristics of the isolates obtained are yet to be described.

Genomic investigations have produced valuable insights into environmentally-acquired *Salmonella* in other countries. For example, a study in neighbouring New Zealand was able to hypothesise that an environmental strain of *S*. Typhimurium was first introduced between 1996–1998, with bidirectional transmission occurring between humans and animal hosts [10]. Other studies have used genomics to describe antimicrobial resistance (AMR) and pathogenicity genes [11, 12].

In this paper we extend the outbreak investigation of *S*. Wangata using whole genome sequencing (WGS) and comparative genomics to characterise and describe the *S*. Wangata isolates recovered from different (human, animal, environment) sources in NSW, Australia. In addition to the 84 isolates obtained during the outbreak investigation, 6 isolates from routine surveillance in NSW and 8 isolates from routine surveillance overseas were included in the analysis. These additional isolates were included to maximise the number of *S*. Wangata samples in the study and to compare isolates from outbreak and non-outbreak origins. Our aim was to understand the diversity of *S*. Wangata isolates circulating during the outbreak period and to determine the relatedness between the isolates associated with human infections and those recovered from other sources in NSW.

## Methods

### *S*. Wangata isolates

Ninety Australian *S*. Wangata isolates were included in this study, 84 of which were obtained during an outbreak investigation conducted between November 2016 and April 2017 [9]. An additional six isolates were included and were obtained from routine surveillance of food (n = 3) and humans (n = 3) in NSW.

Outbreak isolates were cultured from humans (n = 75), wildlife (n = 7), a companion animal (n = 1) and compost (n = 1) and originated from various locations across three Local Health Districts in north-eastern NSW (combined total area of 163, 857 km^2^). Detailed descriptions of the sample collection and isolation methodology are available in our previous publication [9]. Briefly, human isolates were obtained from diagnostic laboratories which used standard protocols for isolating *Salmonella* from clinical (stool) samples. Compost, companion animal (pet dog) and wildlife isolates were obtained from samples collected from the household environment of human cases or from wildlife rehabilitation centres in the study districts. Wildlife and companion animal samples were either faecal samples or cloacal swabs; of the three animals with clinical histories (one pet dog and two of four wildlife animals from rehabilitation centres), none had signs of gastroenteritis at the time of sampling. Isolates from compost, companion animal and wildlife samples were obtained following standard enrichment and growth on selective media. All suspect isolates underwent confirmatory testing and were serotyped according to the White-Kaufmann-Le Minor scheme [13].

The accession numbers and details of the *S*. Wangata isolates used in this study can be found in S1 Table.

### Whole genome sequencing

Genomic DNA was extracted from each *S*. Wangata isolate using a manual extraction kit (Presto^™^ Mini gDNA Bacteria Kit, Geneaid), according to the manufacturer’s instructions. Sequencing libraries with a 150 bp insert size were prepared using a Nextera XT library prep kit and the Index set (Illumina). Libraries were sequenced on an Illumina NextSeq500 platform (Illumina) at the Centre for Infectious Diseases and Microbiology Laboratory Services and the Centre for Infectious Diseases and Microbiology–Public Health (CIDM-PH), NSW Health Pathology at Westmead.

### Phylogenomic analysis

A core single-nucleotide polymorphism (SNP) tree of all *S*. Wangata isolates, all publicly available reference *Salmonella* genomes, and seven draft genomes of serovars of interest was constructed. The serovars of interest consisted of: serovars commonly isolated from human samples in NSW (*S*. Birkenhead, *S*. Bovismorbificans, *S*. Infantis); serovars isolated from wildlife during the environmental investigation of *S*. Wangata (*S*. Birkenhead, *S*. Bovismorbificans, *S*. Chailey, *S*. Kiambu, *S*. Kottbus)[9]; and a serovar in Australia primarily transmitted from environmental sources (*S*. Mississippi). The details of reference genomes are given in S2 Table.

Reads were trimmed using Trimmomatic 0.36 [14] to remove trailing end bases with a phred score <33. Snippy 3.1 (https://github.com/tseemann/snippy) was then used to align the trimmed reads against the core genes of *Salmonella enterica* [15]. The pairwise distances were estimated using the Jukes-Cantor model with FastTree 2.1.9 [16] and a maximum-likelihood SNP tree was constructed. To further investigate the phylogeny within the *S*. Wangata serovar, a second SNP tree comparing just the *S*. Wangata isolates was subsequently constructed using the method described above. The core genome did not provide sufficient discrimination between these isolates, therefore a previously constructed draft genome of *S*. Wangata [9] was used as the reference for the *S*. Wangata phylogeny. Due to the high level of relatedness between the Australian *S*. Wangata isolates, individual SNPs used to develop the *S*. Wangata phylogeny were identified and characterised.

### *In silico* typing

For each *S*. Wangata isolate the multi-locus sequence type (MLST) was extracted from the sequencing data using the MLST 2.0 database [17]. MLST types were then used to determine the eBURST group by searching for the MLST type in the EnteroBase database [18]. *In silico* serotyping was performed using SeqSero 1.2 [19].

### Characterisation

Pathogenicity islands and virulence determinants were identified by mapping sample reads to a concatenated file of all identified pathogenicity regions available from the Pathogenicity Island Database PAIDB 2.0 (www.paidb.re.kr/). Mapped-reads were visually inspected and classed as present, absent or incomplete depending on the coverage of reads mapped to the region.

### Assembly and annotation

The sequencing data of 36 representative *S*. Wangata isolates, recovered from different sources, were assembled *de novo* to generate draft genomes using a local pipeline. Briefly, paired-end reads were trimmed using Trimmomatic [14] and the trimmed reads were assembled using Velvet v0.7.03 [20]. The parameters were optimised to give the best Kmer size and at least 30x coverage of each Kmer using VelvetOptimiser. The assembled genomes were annotated using Prokka [21].

### Comparative genomics

Comparisons between individual draft genomes were performed using TBLASTX [22] and were viewed in the Artemis Comparison Tool (ACT) for manual comparison of the genomes [23].

The assembled *S*. Wangata genomes were further characterised. *Salmonella* pathogenicity island types were identified *in silico* using and SPIFinder 1.0 (https://cge.cb*s*.dtu.dk/services/SPIFinder/) and AMR genotypes and chromosomal point mutations associated with resistance were identified using the Comprehensive Antibiotic Resistance Database (CARD) [24]. Assembled genomes were additionally scanned for the presence of prophages using PHASTER [25]. The presence/absence of virulence and resistance determinants were compared between assemblies.

### Ethical approval

Human ethics approval was obtained from the Hunter New England Local Health District Human Research Ethics Committee (LNR/16/HNE/485, granted 1 November 2016) and the Australian National University (2016/605, granted 2 December 2016). Animal ethics was obtained from the University of Sydney (2016/1076, granted 16 November 2016).

## Results

### Phylogenomic analysis of *Salmonella* Wangata isolates

**Fig 1:**
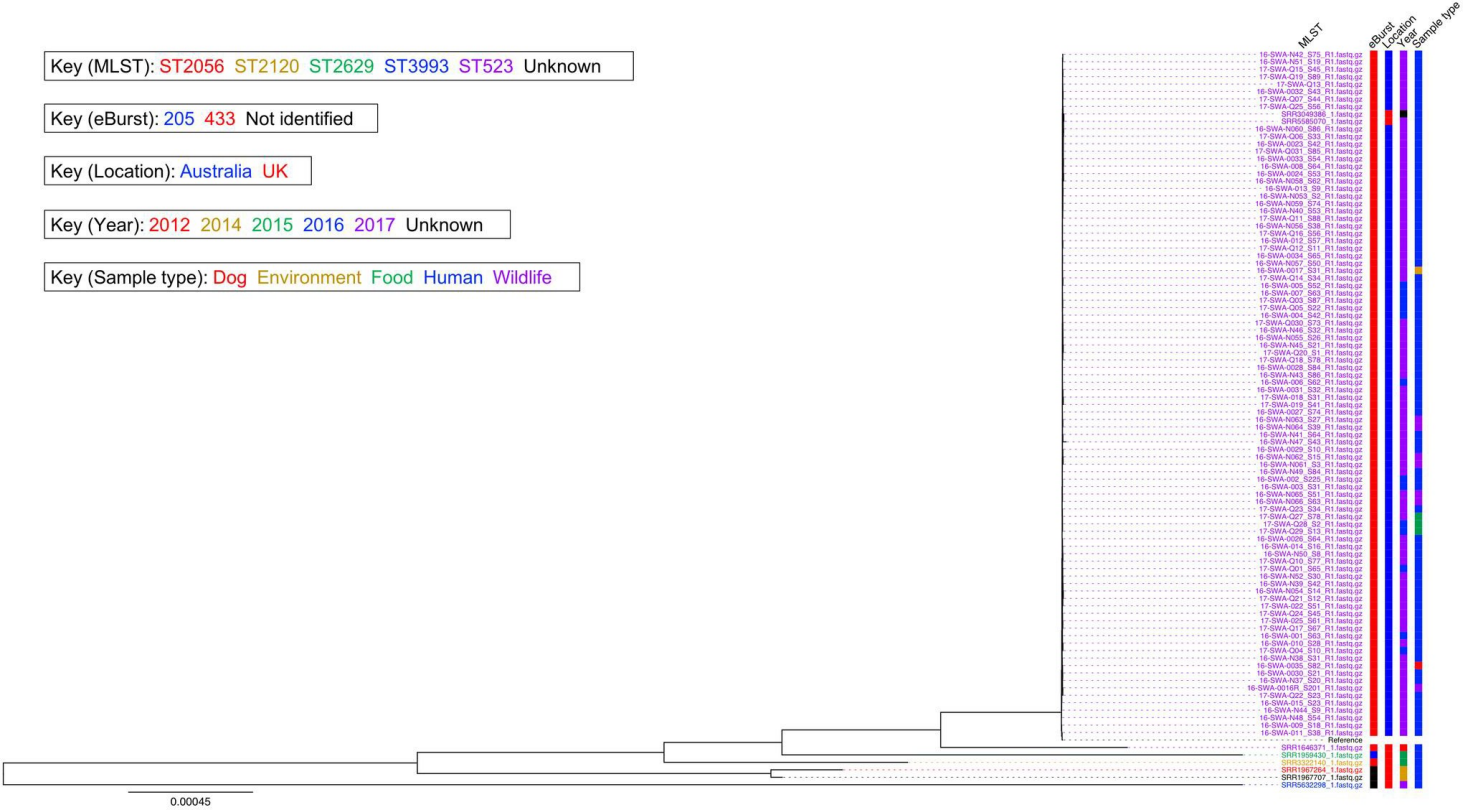
Phylogeny of all *S*. Wangata isolates mapped against the draft *S*. Wangata reference genome. Labels are coloured according to MLST type, eBurst group, location, year of sample collection and sample type. One isolate (SRR1967707) had an unknown MLST type (indicated in black). The eBurst group was not identified for three UK isolates (indicated in black) and year was not known for one UK isolate (indicated in black).

The phylogeny of the *S*. Wangata isolates is shown in Fig 1. Isolates were clustered by MLST type, with all Australian isolates (ST523) clustering together. There was no clustering by sample type or year. Isolates were partially clustered by location with six of the eight UK isolates occurring outside of the main clade. The two remaining UK isolates were located within the predominantly Australian clade. The phylogenetic tree of all available reference *Salmonella* genomes and *S*. Wangata isolates is shown in S1 Fig.

The median SNP distance between the Australian outbreak and non-outbreak *S*. Wangata isolates was 24 SNPs (range 5–103 SNPs). The two homologous UK isolates were also separated from the Australian isolates by a median of 24 SNPs each. By comparison, the six remaining UK isolates had a much larger distance from the Australian isolates, with a maximum SNP difference of between 5137 and 31,072 SNPs. Details of the SNPs in the *S*. Wangata isolates are given in S2 Fig.

### Characterisation and comparison of *S*. Wangata

All Australian isolates displayed identical pathogenicity profiles as indicated by the mapping of reads to genes and regions identified in the Pathogenicity Island Database (S3 Fig). There was no variation between the Australian isolates with regards to the presence, absence or partial mapping of reads. There was, however, a degree of variation in the UK isolates, particularly in relation to SPI-5 where regions that were partially mapped by reads from Australian isolates were absent in a number of the UK isolates. Similarly, reads from one UK isolate (SRR5632298) mapped against a partial SPI-10 sequence which was absent from the Australian isolates.

**Fig 2:**
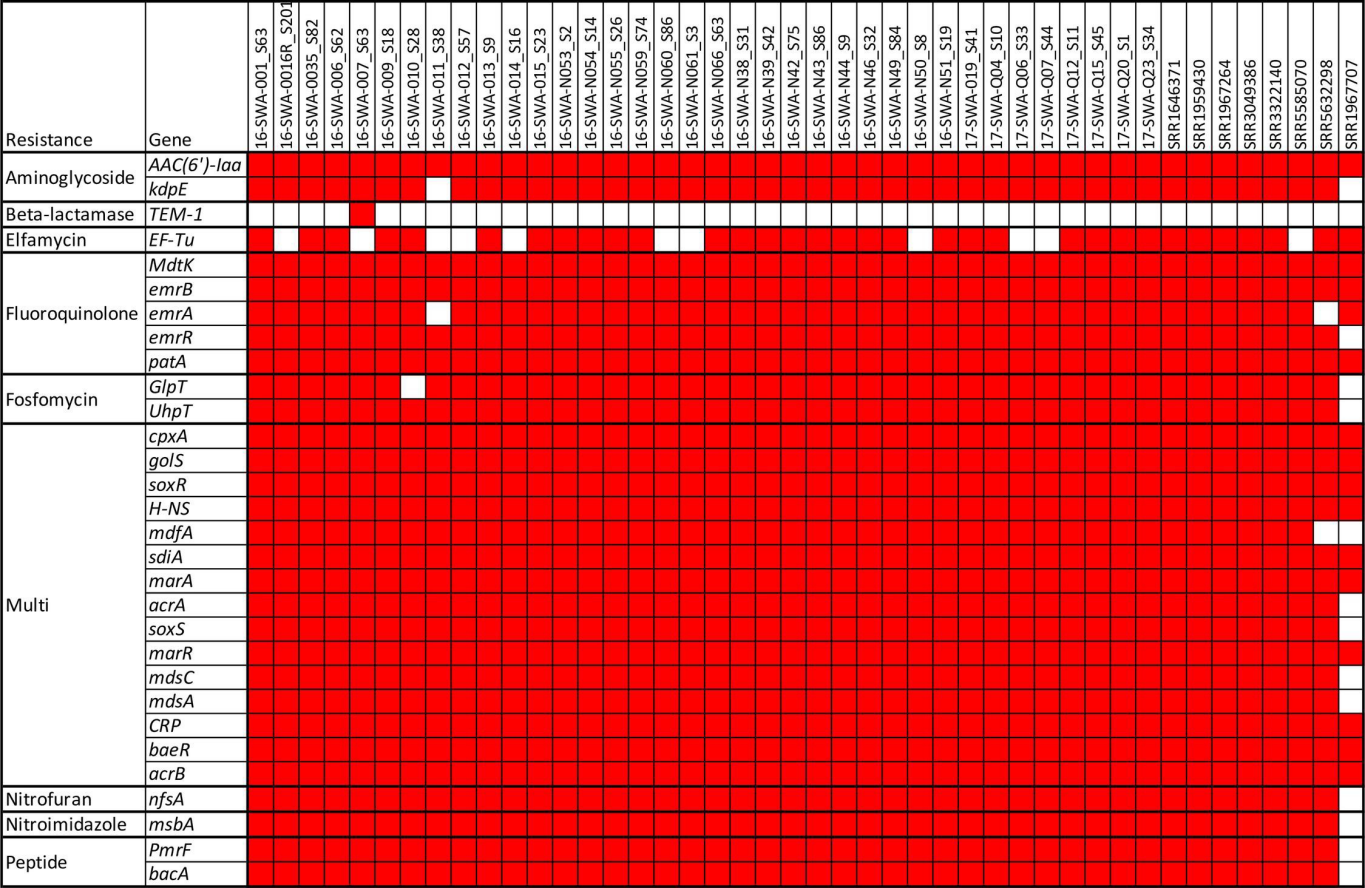
Heatmap of resistance genes identified using Resfinder and CARD. Genes are indicated as present (red) or absent (white). The threshold for identification was taken to be 80% gene ID and 50% sequence length.

A representative selection of isolates was assembled (n = 44). Comparison of isolates from different locations and sample types revealed no major genomic differences between isolates obtained from humans, animals or the environment. Characterisation of assembled genomes revealed identical SPI and virulence architectures. Identical SPI regions were identified in all assembled genomes; namely; SPI-1, SPI-2, SPI-3, SPI-4, SPI-5, SPI-9, SPI-11, SPI-12, SPI-13, SPI-14, SPI-16 and C63P1. A similar AMR profile was also detected in all assembled *S*. Wangata isolates (Fig 2). Overall, 30 resistance genes were detected across the 44 assembled isolates. Over half the genes detected (16/30) were associated with resistance to multiple antibiotics. Thirty-one isolates possessed identical patterns (29 resistance genes, including all 16 genes associated with multi-resistance). Remaining isolates variably lacked the *EF-Tu* (n = 11), *emrA* (n = 2), *kdpE* (n = 2), *GlpT* (n = 1) and *mdfA* 9 (n = 1) genes. One UK isolate (SRR1967707) lacked additional resistance genes. *TEM-1*, a beta-lactamase, was only detected in one isolate (16-SWA-007). Collectively, the genes detected predict reduced susceptibility to 22 classifications of antimicrobials.

PHASTER identified one prophage, Gifsy-2, in 34 of the 36 assembled Australian *S*. Wangata isolates. One of the isolates had Gifsy-2 detected but was classified as ‘questionable’ (completeness score 70–90). Seven isolates also had prophage 186 intact and prophage P4 classified as ‘questionable’. Gifsy-1 was identified in one isolate (17-SWA-Q23). Investigation of the virulence genes associated with Gifsy-2 identified *sodC1* but not *gtgA*.

Sequences described in this study can be found under the study accession number PRJEB30345. Individual accession numbers are provided in S1 Table.

## Discussion

*S*. Wangata is an emerging serovar in NSW, Australia and is hypothesised to be transmitted via a non-foodborne pathway/environmental route. Using isolates obtained during the investigation of a slow moving outbreak and routine surveillance, we present the first genomic investigation of this serovar and describe its associated virulence and AMR characteristics.

Our analysis supports the hypothesis generated during the epidemiological outbreak investigation that *S*. Wangata in NSW is being exchanged between humans, animals and the environment [9]. Isolates of *S*. Wangata obtained from a variety of sources and locations within NSW were all phylogenetically clustered and had the same MLST type. Comparison of assembled genomes revealed there were no major genotypic distinctions between isolates obtained from humans, animals, food or the environment. Furthermore, these isolates also shared highly similar virulence and AMR characteristics. These findings are consistent with genomic investigations of other serovars overseas that suggested *Salmonella* serovars were being transmitted bidirectionally between humans and wildlife [10, 26]. Results from this study are not conclusive regarding the direction of transmission and continued research characterising transmission is warranted given the increasing incidence in humans.

An unexpected finding from this study is the clustering of two UK *S*. Wangata isolates within the Australian *S*. Wangata clade. Of the 78 Australian human isolates, 48 (62%) are known to have not travelled more than 100km from their home in the 7 days prior to illness onset, further supporting the hypothesis of a local acquisition of infection. While it cannot be ruled out that the UK isolates were acquired from overseas sources, given the comparatively small average SNP distance (24 SNPs) of these isolates to the Australian isolates it is also possible that these cases are indicative of travel to Australia. Epidemiological data from these cases is required to confirm this hypothesi*s*.

Antimicrobial resistance is a major concern in Australia [27]. Spill over of AMR genes from domestic sources to wildlife has been observed in a number of studies [28, 29] and is a relevant concern for *S*. Wangata given the strong association with a wildlife or environmental reservoir. While this study revealed a number of genes associated with AMR in the Australian *S*. Wangata isolates, it is important to make the distinction that genotypic resistance may not be indicative of phenotypic resistance. Fluoroquinolones are important for the treatment of salmonellosis yet prevalence surveys indicate the rate of fluoroquinolone resistance in Australia is low in both *Salmonella* and other enteric bacteria [30, 31]. Similarly, aminoglycoside resistance has been observed in other serovars in Australia but isolates are generally uncommon [30]. The identification of genes associated with resistance to these antimicrobials was therefore unexpected. Nevertheless, the majority of the genes identified in the *S*. Wangata isolates code for efflux pumps. Efflux pumps are ubiquitous in bacterial genomes and AMR is associated with select efflux pumps when overexpressed [32]. We did not investigate the expression of these genes so cannot conclude if *S*. Wangata was phenotypically resistant. Further research that includes phenotypic testing of isolates is required to determine if *S*. Wangata has a decreased sensitivity to antimicrobials.

SPI-1 and SPI-2 are highly conserved regions within the *Salmonella* genome [33] and are typically associated with gut wall invasion and intracellular survival and replication, respectively [34]. We did not identify any novel pathogenicity genes within the genome of *S*. Wangata. The clinical virulence of *S*. Wangata is unknown however the epidemiological arm of the outbreak investigation found 42% of cases required hospitalisation, although this was not statistically significantly different when compared to control cases of *S*. Typhimurium [9]. A study investigating invasive and non-invasive *S*. Dublin failed to define the precise genomic mechanism of virulence, instead concluding that host immunity might play a significant role [35]. This might also be the case with *S*. Wangata and highlights the need for future studies to include detailed clinical history of cases. Increased pathogenicity has also been associated with increased host specificity [36]. However, our results indicate that *S*. Wangata occurs in a number of host species suggesting this is not a factor in *S*. Wangata pathogenesis.

The prophage Gifsy-2 is widely disseminated throughout many *Salmonella* strains [37] and has been associated with increased virulence in *S*. Typhimurium [38] and *S*. Dublin [35]. In particular, the presence of the s*odC1* gene, observed in the *S*. Wangata isolates, is believed to protect the bacteria from superoxide produced by macrophages [39]. While these genomic components may play a role in the pathogenesis of *S*. Wangata, detailed clinical history of patients is required to determine the true virulent nature of *S*. Wangata.

There were a number of limitations in our study. We were not able to confirm the *in silico* resistance results with phenotypic testing of antimicrobial resistance. Other studies have found *in silico* results conform to phenotypic testing and have a high sensitivity and specificity [26, 40] although specificity may vary depending on the type of antibiotic being tested [40]. In particular, confirmation of phenotypic fluoroquinolone resistance should be undertaken given the low prevalence typically observed of this phenotype in Australia. Detailed clinical histories of the human cases were not available. This may have helped guide and interpret the importance of identified pathogenicity genes. Similarly, epidemiological data are required to support the hypothesis of travel related dispersion of *S*. Wangata.

Results from this study support epidemiological evidence generated during the outbreak investigation that *S*. Wangata is shared by humans, domestic animals, wildlife and the environment. The presence of AMR and pathogenicity genes highlights the potential clinical importance of *S*. Wangata however further research is required to determine the relevance of identified genomic characteristics. We demonstrate that the use of WGS is a powerful tool in characterising a poorly known serovar. Data presented here can be used as a basis for ongoing surveillance of *S*. Wangata in NSW.

## Supporting information

S1 Table Detailed list of *S*. Wangata isolates used in this study. (DOCX)

S2 Table Detailed list of all references *Salmonella* genomes used in this study. (XLSX)

S1 Fig Phylogenetic tree of all publicly available reference *Salmonella* genomes, all *S*. Wangata isolates, and seven draft genomes of serovars of interest. The S. Wangata cluster (shaded in orange) is comprised of all *S*. Wangata isolates and *S*. Slotedijk (ATCC 15791).
(PDF)

S2 Fig SNP differences observed between *S*. Wangata isolates. (DOCX)

S3 Fig Heatmap of pathogenicity islands in the Australian *S*. Wangata isolates. Pathogenicity islands were extracted from the Pathogenicity Island Database PAIDB 2.0 (www.paidb.re.kr/). Regions are defined as present (red), absent (white) or incomplete (pink) as determined by visual inspection of the coverage of reads mapped to the region.
(TIF)
