# Supplementary material for: Genomic characterisation of Salmonella enterica serovar Wangata isolates obtained from different sources reveals low genomic diversity
Source: PLoS One. 2020 Feb 28;15(2):e0229697. doi: 10.1371/journal.pone.0229697 (PMC7048276; doi:10.1371/journal.pone.0229697)
Supplement: S1 Table — (DOCX) [file pone.0229697.s001.docx]

# S1 Table: Detailed list of *S*. Wangata isolates used in this study

| **Country** | **Collected by** | **Sample ID** | **Month collected** | **Sample accession no.** | **Host** | **MLST** | **% reads Phred** ≥ **35** | **% reads with no N bases** | **No. Contigs** | **N50** |
| --- | --- | --- | --- | --- | --- | --- | --- | --- | --- | --- |
| Australia | Outbreak investigation | 16-SWA-001_S63* | Nov-16 | ERS2983170 | Human | ST-523 | 14.68 | 99.72 | 96 | 187530 |
| Australia | Outbreak investigation | 16-SWA-0016R_S201* | Jan-17 | ERS2983171 | Wildlife (unknown) | ST-523 | 39.75 | 99.73 | 103 | 187530 |
| Australia | Outbreak investigation | 16-SWA-0017_S31 | Jan-17 | ERS2983172 | Compost | ST-523 | 15.89 | 99.55 |  |  |
| Australia | Outbreak investigation | 16-SWA-002_S225 | Dec-16 | ERS2983173 | Human | ST-523 | 3.96 | 99.69 |  |  |
| Australia | Outbreak investigation | 16-SWA-0023_S42 | Feb-17 | ERS2983174 | Human | ST-523 | 21.82 | 99.55 |  |  |
| Australia | Outbreak investigation | 16-SWA-0024_S53 | Feb-17 | ERS2983175 | Human | ST-523 | 19.82 | 99.55 |  |  |
| Australia | Outbreak investigation | 16-SWA-0026_S64 | Mar-17 | ERS2983176 | Human | ST-523 | 14.17 | 99.57 |  |  |
| Australia | Outbreak investigation | 16-SWA-0027_S74 | Mar-17 | ERS2983177 | Human | ST-523 | 17.17 | 99.57 |  |  |
| Australia | Outbreak investigation | 16-SWA-0028_S84 | Feb-17 | ERS2983178 | Human | ST-523 | 15.53 | 99.57 |  |  |
| Australia | Outbreak investigation | 16-SWA-0029_S10 | Feb-17 | ERS2983179 | Human | ST-523 | 21.65 | 99.56 |  |  |
| Australia | Outbreak investigation | 16-SWA-003_S31 | Dec-16 | ERS2983180 | Human | ST-523 | 38.39 | 99.78 |  |  |
| Australia | Outbreak investigation | 16-SWA-0030_S21 | Feb-17 | ERS2983181 | Human | ST-523 | 14.26 | 99.58 |  |  |
| Australia | Outbreak investigation | 16-SWA-0031_S32 | Feb-17 | ERS2983182 | Human | ST-523 | 20.55 | 99.56 |  |  |
| Australia | Outbreak investigation | 16-SWA-0032_S43 | Feb-17 | ERS2983183 | Human | ST-523 | 18.73 | 99.57 |  |  |
| Australia | Outbreak investigation | 16-SWA-0033_S54 | Feb-17 | ERS2983184 | Human | ST-523 | 22.76 | 99.55 |  |  |
| Australia | Outbreak investigation | 16-SWA-0034_S65 | Feb-17 | ERS2983185 | Human | ST-523 | 17.61 | 99.59 |  |  |
| Australia | Outbreak investigation | 16-SWA-0035_S82* | Mar-17 | ERS2983186 | Dog | ST-523 | 18.17 | 99.87 | 102 | 138122 |
| Australia | Outbreak investigation | 16-SWA-004_S42 | Dec-16 | ERS2983187 | Human | ST-523 | 32.23 | 99.79 |  |  |
| Australia | Outbreak investigation | 16-SWA-005_S52 | Dec-16 | ERS2983188 | Human | ST-523 | 26.29 | 99.79 |  |  |
| Australia | Outbreak investigation | 16-SWA-006_S62* | Dec-16 | ERS2983189 | Human | ST-523 | 28.69 | 99.80 | 98 | 176289 |
| Australia | Outbreak investigation | 16-SWA-007_S63* | Dec-16 | ERS2983190 | Human | ST-523 | 25.20 | 99.80 | 98 | 200883 |
| Australia | Outbreak investigation | 16-SWA-008_S64 | Jan-17 | ERS2983191 | Human | ST-523 | 23.35 | 99.80 |  |  |
| Australia | Outbreak investigation | 16-SWA-009_S18* | Jan-17 | ERS2983192 | Human | ST-523 | 8.26 | 99.70 | 100 | 143033 |
| Australia | Outbreak investigation | 16-SWA-010_S28* | Jan-17 | ERS2983193 | Human | ST-523 | 11.06 | 99.70 | 207 | 55127 |
| Australia | Outbreak investigation | 16-SWA-011_S38* | Jan-17 | ERS2983194 | Human | ST-523 | 6.03 | 99.85 | 795 | 12206 |
| Australia | Outbreak investigation | 16-SWA-012_S57* | Jan-17 | ERS2983195 | Human | ST-523 | 3.87 | 99.90 | 139 | 108189 |
| Australia | Outbreak investigation | 16-SWA-013_S9* | Jan-17 | ERS2983196 | Human | ST-523 | 5.49 | 99.90 | 122 | 142952 |
| Australia | Outbreak investigation | 16-SWA-014_S16* | Jan-17 | ERS2983197 | Human | ST-523 | 6.36 | 99.91 | 111 | 137881 |
| Australia | Outbreak investigation | 16-SWA-015_S23* | Jan-17 | ERS2983198 | Human | ST-523 | 5.61 | 99.92 | 142 | 110362 |
| Australia | Outbreak investigation | 16-SWA-N053_S2* | Apr-17 | ERS2983199 | Human | ST-523 | 21.49 | 99.67 | 98 | 170884 |
| Australia | Outbreak investigation | 16-SWA-N054_S14* | Apr-17 | ERS2983200 | Human | ST-523 | 22.60 | 99.66 | 100 | 178870 |
| Australia | Outbreak investigation | 16-SWA-N055_S26* | Apr-17 | ERS2983201 | Human | ST-523 | 23.09 | 99.66 | 259 | 57469 |
| Australia | Outbreak investigation | 16-SWA-N056_S38 | Apr-17 | ERS2983202 | Human | ST-523 | 25.51 | 99.63 |  |  |
| Australia | Outbreak investigation | 16-SWA-N057_S50 | Apr-17 | ERS2983203 | Human | ST-523 | 27.30 | 99.65 |  |  |
| Australia | Outbreak investigation | 16-SWA-N058_S62 | Apr-17 | ERS2983204 | Human | ST-523 | 25.40 | 99.67 |  |  |
| Australia | Outbreak investigation | 16-SWA-N059_S74* | Apr-17 | ERS2983205 | Human | ST-523 | 22.54 | 99.69 | 111 | 170895 |
| Australia | Outbreak investigation | 16-SWA-N060_S86* | Apr-17 | ERS2983206 | Human | ST-523 | 16.57 | 99.69 | 103 | 200654 |
| Australia | Outbreak investigation | 16-SWA-N061_S3* | May-17 | ERS2983207 | Turtle | ST-523 | 21.45 | 99.67 | 103 | 170884 |
| Australia | Outbreak investigation | 16-SWA-N062_S15 | May-17 | ERS2983208 | Turtle | ST-523 | 20.34 | 99.68 |  |  |
| Australia | Outbreak investigation | 16-SWA-N063_S27 | Apr-17 | ERS2983209 | Pelican | ST-523 | 18.54 | 99.68 |  |  |
| Australia | Outbreak investigation | 16-SWA-N064_S39 | Apr-17 | ERS2983210 | Swan | ST-523 | 23.96 | 99.66 |  |  |
| Australia | Outbreak investigation | 16-SWA-N065_S51 | Apr-17 | ERS2983211 | Wildlife (unknown) | ST-523 | 27.11 | 99.66 |  |  |
| Australia | Outbreak investigation | 16-SWA-N066_S63* | Apr-17 | ERS2983212 | Wildlife (unknown) | ST-523 | 19.32 | 99.66 | 101 | 144154 |
| Australia | Outbreak investigation | 16-SWA-N37_S20 | Apr-17 | ERS2983213 | Human | ST-523 | 11.34 | 99.81 |  |  |
| Australia | Outbreak investigation | 16-SWA-N38_S31* | Apr-17 | ERS2983214 | Human | ST-523 | 10.05 | 99.82 | 100 | 170894 |
| Australia | Outbreak investigation | 16-SWA-N39_S42* | Apr-17 | ERS2983215 | Human | ST-523 | 10.90 | 99.82 | 95 | 214117 |
| Australia | Outbreak investigation | 16-SWA-N40_S53 | Mar-17 | ERS2983216 | Human | ST-523 | 11.82 | 99.82 |  |  |
| Australia | Outbreak investigation | 16-SWA-N41_S64 | Mar-17 | ERS2983217 | Human | ST-523 | 12.04 | 99.82 |  |  |
| Australia | Outbreak investigation | 16-SWA-N42_S75* | Mar-17 | ERS2983218 | Human | ST-523 | 10.34 | 99.80 | 108 | 176213 |
| Australia | Outbreak investigation | 16-SWA-N43_S86* | Mar-17 | ERS2983219 | Human | ST-523 | 10.12 | 99.82 | 99 | 189430 |
| Australia | Outbreak investigation | 16-SWA-N44_S9* | Mar-17 | ERS2983220 | Human | ST-523 | 9.90 | 99.83 | 114 | 142944 |
| Australia | Outbreak investigation | 16-SWA-N45_S21 | Mar-17 | ERS2983221 | Human | ST-523 | 10.56 | 99.82 |  |  |
| Australia | Outbreak investigation | 16-SWA-N46_S32* | Mar-17 | ERS2983222 | Human | ST-523 | 10.72 | 99.82 | 105 | 144351 |
| Australia | Outbreak investigation | 16-SWA-N47_S43 | Mar-17 | ERS2983223 | Human | ST-523 | 9.67 | 99.82 |  |  |
| Australia | Outbreak investigation | 16-SWA-N48_S54 | Apr-17 | ERS2983224 | Human | ST-523 | 11.46 | 99.81 |  |  |
| Australia | Outbreak investigation | 16-SWA-N49_S84* | Mar-17 | ERS2983225 | Human | ST-523 | 13.27 | 99.83 | 114 | 170884 |
| Australia | Outbreak investigation | 16-SWA-N50_S8* | Apr-17 | ERS2983226 | Human | ST-523 | 11.67 | 99.83 | 111 | 175712 |
| Australia | Outbreak investigation | 16-SWA-N51_S19* | Apr-17 | ERS2983227 | Human | ST-523 | 14.11 | 99.81 | 97 | 170897 |
| Australia | Outbreak investigation | 16-SWA-N52_S30 | Apr-17 | ERS2983228 | Human | ST-523 | 15.21 | 99.82 |  |  |
| Australia | Outbreak investigation | 17-SWA-018_S31 | Mar-17 | ERS2983229 | Human | ST-523 | 9.66 | 99.76 |  |  |
| Australia | Outbreak investigation | 17-SWA-019_S41* | Mar-17 | ERS2983230 | Human | ST-523 | 7.85 | 99.73 | 111 | 152026 |
| Australia | Outbreak investigation | 17-SWA-022_S51 | Mar-17 | ERS2983231 | Human | ST-523 | 10.28 | 99.75 |  |  |
| Australia | Outbreak investigation | 17-SWA-025_S61 | Feb-17 | ERS2983232 | Human | ST-523 | 11.07 | 99.74 |  |  |
| Australia | Routine surveillance | 17-SWA-Q01_S65 | Oct-16 | ERS2983233 | Human | ST-523 | 13.00 | 99.81 |  |  |
| Australia | Outbreak investigation | 17-SWA-Q03_S87 | Dec-16 | ERS2983234 | Human | ST-523 | 10.10 | 99.83 |  |  |
| Australia | Outbreak investigation | 17-SWA-Q030_S73 | Apr-17 | ERS2983235 | Human | ST-523 | 20.99 | 99.67 |  |  |
| Australia | Outbreak investigation | 17-SWA-Q031_S85 | Apr-17 | ERS2983236 | Human | ST-523 | 19.99 | 99.68 |  |  |
| Australia | Outbreak investigation | 17-SWA-Q04_S10* | Dec-16 | ERS2983237 | Human | ST-523 | 9.98 | 99.83 | 113 | 138019 |
| Australia | Outbreak investigation | 17-SWA-Q05_S22 | Dec-16 | ERS2983238 | Human | ST-523 | 12.25 | 99.82 |  |  |
| Australia | Outbreak investigation | 17-SWA-Q06_S33* | Jan-17 | ERS2983239 | Human | ST-523 | 10.77 | 99.82 | 96 | 151663 |
| Australia | Outbreak investigation | 17-SWA-Q07_S44* | Jan-17 | ERS2983240 | Human | ST-523 | 11.04 | 99.83 | 96 | 187420 |
| Australia | Outbreak investigation | 17-SWA-Q10_S77 | Jan-17 | ERS2983241 | Human | ST-523 | 11.68 | 99.81 |  |  |
| Australia | Outbreak investigation | 17-SWA-Q11_S88 | Feb-17 | ERS2983242 | Human | ST-523 | 11.88 | 99.80 |  |  |
| Australia | Outbreak investigation | 17-SWA-Q12_S11* | Feb-17 | ERS2983243 | Human | ST-523 | 8.04 | 99.84 | 106 | 170844 |
| Australia | Outbreak investigation | 17-SWA-Q13 | Feb-17 | ERS2983244 | Human | ST-523 | 12.06 | 99.82 |  |  |
| Australia | Outbreak investigation | 17-SWA-Q14_S34 | Feb-17 | ERS2983245 | Human | ST-523 | 10.97 | 99.83 |  |  |
| Australia | Outbreak investigation | 17-SWA-Q15_S45* | Feb-17 | ERS2983246 | Human | ST-523 | 10.41 | 99.81 | 105 | 143868 |
| Australia | Outbreak investigation | 17-SWA-Q16_S56 | Feb-17 | ERS2983247 | Human | ST-523 | 11.89 | 99.81 |  |  |
| Australia | Outbreak investigation | 17-SWA-Q17_S67 | Mar-17 | ERS2983248 | Human | ST-523 | 12.51 | 99.81 |  |  |
| Australia | Outbreak investigation | 17-SWA-Q18_S78 | Mar-17 | ERS2983249 | Human | ST-523 | 9.69 | 99.81 |  |  |
| Australia | Outbreak investigation | 17-SWA-Q19_S89 | Mar-17 | ERS2983250 | Human | ST-523 | 10.18 | 99.80 |  |  |
| Australia | Outbreak investigation | 17-SWA-Q20_S1* | Mar-17 | ERS2983251 | Human | ST-523 | 10.91 | 99.82 | 109 | 170885 |
| Australia | Outbreak investigation | 17-SWA-Q21_S12 | Mar-17 | ERS2983252 | Human | ST-523 | 13.73 | 99.82 |  |  |
| Australia | Outbreak investigation | 17-SWA-Q22_S23 | Mar-17 | ERS2983253 | Human | ST-523 | 14.81 | 99.82 |  |  |
| Australia | Outbreak investigation | 17-SWA-Q23_S34* | Mar-17 | ERS2983254 | Human | ST-523 | 11.71 | 99.83 | 119 | 168699 |
| Australia | Outbreak investigation | 17-SWA-Q24_S45 | Mar-17 | ERS2983255 | Human | ST-523 | 14.70 | 99.82 |  |  |
| Australia | Outbreak investigation | 17-SWA-Q25_S56 | Mar-17 | ERS2983256 | Human | ST-523 | 14.47 | 99.82 |  |  |
| Australia | Routine surveillance | 17-SWA-Q27_S78 | N/A | ERS2983257 | Macadamia | ST-523 | 13.04 | 99.82 |  |  |
| Australia | Routine surveillance | 17-SWA-Q28_S2 | N/A | ERS2983258 | Macadamia | ST-523 | 11.09 | 99.83 |  |  |
| Australia | Routine surveillance | 17-SWA-Q29_S13 | N/A | ERS2983259 | Macadamia | ST-523 | 13.44 | 99.81 |  |  |
| UK | Public Health England | SRR1646371* | Jun-12 | SRR1646371 | Human | ST-523 |  |  | 249 | 47080 |
| UK | Public Health England | SRR1959430* | Jan-15 | SRR1959430 | Human | ST-2629 |  |  | 116 | 112007 |
| UK | Public Health England | SRR1967264* | Aug-14 | SRR1967264 | Human | ST-2056 |  |  | 88 | 170625 |
| UK | Public Health England | SRR1967707* | Aug-14 | SRR1967707 | Human | Unknown |  |  | 2412 | 3654 |
| UK | Public Health England | SRR3049386* | N/A | SRR3049386 | Human | ST-523 |  |  | 156 | 97121 |
| UK | Public Health England | SRR3322140* | Aug-15 | SRR3322140 | Human | ST-2120 |  |  | 200 | 60098 |
| UK | Public Health England | SRR5585070* | Jan-17 | SRR5585070 | Human | ST-523 |  |  | 109 | 187430 |
| UK | Public Health England | SRR5632298* | Mar-17 | SRR5632298 | Human | ST-3993 |  |  | 922 | 10074 |

* Isolates was assembled.
