# Supplementary material for: Genomic characterisation of Salmonella enterica serovar Wangata isolates obtained from different sources reveals low genomic diversity
Source: PLoS One. 2020 Feb 28;15(2):e0229697. doi: 10.1371/journal.pone.0229697 (PMC7048276; doi:10.1371/journal.pone.0229697)

S3 Fig: Phylogenetic tree of all publicly available reference Salmonella genomes, all S. Wangata isolates, and seven draft genomes of serovars of interest. The S. Wangata cluster (shaded in orange) is comprised of all S. Wangata isolates and S. Slotedijk (ATCC 15791)

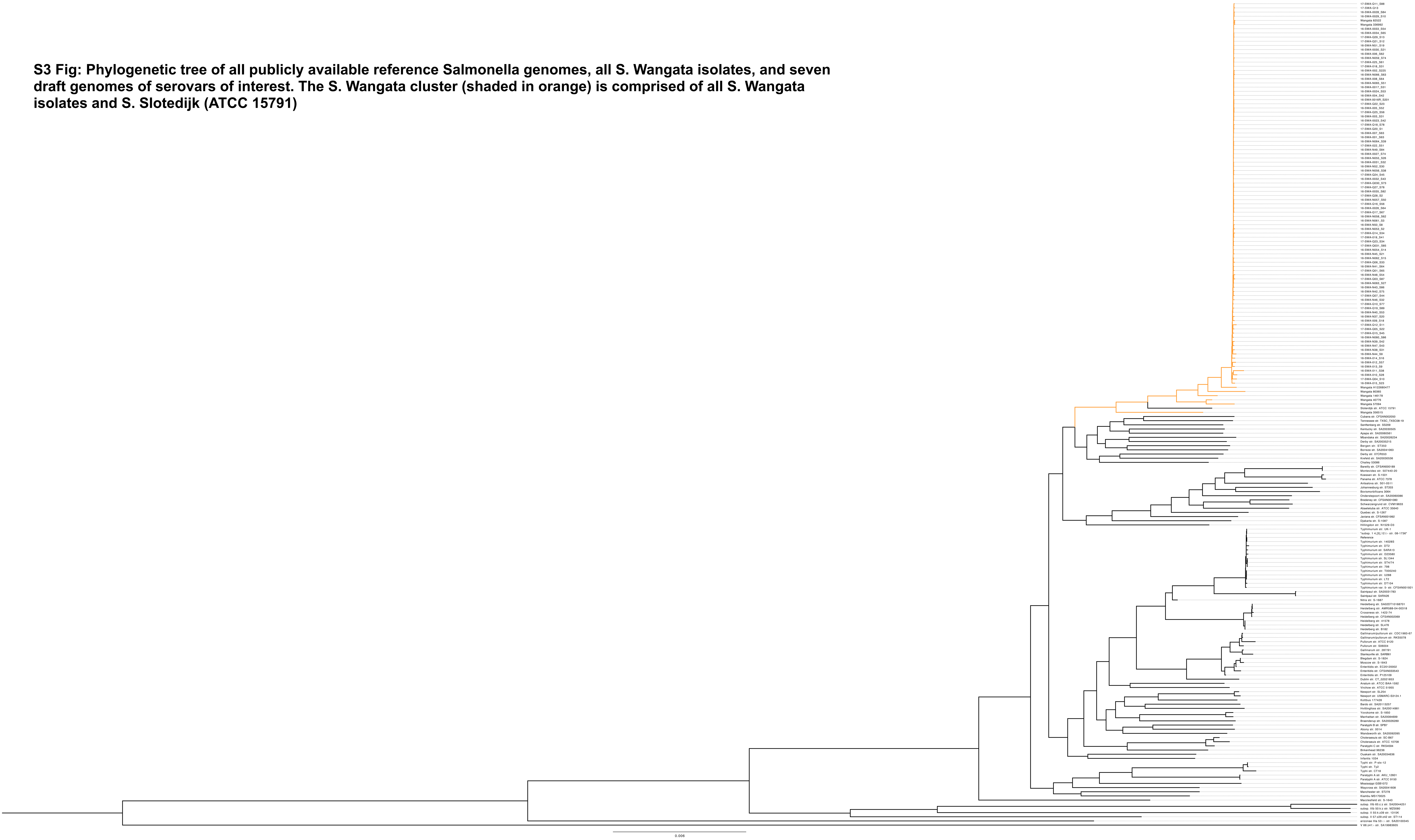

Supplement: S1 Fig — The S. Wangata cluster (shaded in orange) is comprised of all S. Wangata isolates and S. Slotedijk (ATCC 15791). (PDF) [file pone.0229697.s003.pdf]
