# Supplementary material for: Genomic characterisation of Salmonella enterica serovar Wangata isolates obtained from different sources reveals low genomic diversity
Source: PLoS One. 2020 Feb 28;15(2):e0229697. doi: 10.1371/journal.pone.0229697 (PMC7048276; doi:10.1371/journal.pone.0229697)
Supplement: S2 Fig — (DOCX) [file pone.0229697.s004.docx]

Fig S4.1: Details of SNPs observed in at least four of the Australian S. Wangata isolates

| POS | 248500 | 283938 | 433414 | 786026 | 1267327 | 1277426 | 1521839 | 1663573 | 1735968 | 1884609 | 2034437 | 2447181 | 3086281 | 3167398 | 3519795 | 3560772 | 3666065 | 3666067 |
| --- | --- | --- | --- | --- | --- | --- | --- | --- | --- | --- | --- | --- | --- | --- | --- | --- | --- | --- |
| Gene | *yhhQ* | *gntK* | *yedZ* | *papD_1* | *acrD* | *SBOV25411* | *sspH2_1* | *wcaC* | *yvqK* | Unknown | *ydiI* | *hns* | *kdpD* | *citC* | *wapA_6* | *yaeE* | *aceF_2* | *aceF_2* |
| Reference | **T** | **G** | **C** | **G** | **C** | **C** | **A** | **C** | **G** | **C** | **G** | **G** | **A** | **C** | **C** | **G** | **C** | **G** |
| 16-SWA-N42_S75_R1.fastq.gz | - | - | - | - | - | - | - | - | - | - | - | - | - | - | - | - | - | - |
| 16-SWA-N51_S19_R1.fastq.gz | - | - | - | - | - | - | - | - | - | - | - | - | - | - | - | - | - | - |
| 17-SWA-Q16_S56_R1.fastq.gz | - | - | - | - | - | - | - | - | - | - | - | - | - | - | - | - | - | - |
| 16-SWA-N056_S38_R1.fastq.gz | - | - | - | - | - | - | - | - | - | - | - | - | - | - | - | - | - | - |
| 17-SWA-Q19_S89_R1.fastq.gz | G | - | - | - | - | - | - | - | - | T | - | - | - | T | - | - | A | T |
| 17-SWA-Q15_S45_R1.fastq.gz | G | - | - | - | - | - | - | - | - | T | - | - | - | T | - | - | - | - |
| 17-SWA-Q13_R1.fastq.gz | G | - | - | - | - | - | - | - | - | T | - | - | - | T | - | - | - | - |
| 17-SWA-Q07_S44_R1.fastq.gz | G | - | - | - | - | - | - | - | - | T | - | - | - | T | - | - | - | - |
| 16-SWA-0032_S43_R1.fastq.gz | G | - | - | - | - | - | - | - | - | T | - | - | - | T | - | - | - | - |
| 17-SWA-Q11_S88_R1.fastq.gz | G | - | - | - | - | - | - | - | - | T | - | - | - | T | - | - | - | - |
| 16-SWA-N40_S53_R1.fastq.gz | G | - | - | - | - | - | - | - | - | T | - | - | - | T | - | - | - | - |
| 16-SWA-N059_S74_R1.fastq.gz | G | - | - | - | - | - | - | - | - | T | - | - | - | T | - | - | - | - |
| 16-SWA-013_S9_R1.fastq.gz | G | - | - | - | - | - | - | - | - | T | - | - | - | T | - | - | - | - |
| 16-SWA-N053_S2_R1.fastq.gz | G | - | - | - | - | - | - | - | - | T | - | - | - | T | - | - | - | - |
| 17-SWA-Q25_S56_R1.fastq.gz | G | - | - | - | - | - | - | - | - | T | - | - | - | T | - | - | - | - |
| 16-SWA-0033_S54_R1.fastq.gz | G | - | - | - | - | - | - | - | - | T | - | - | - | T | - | - | - | - |
| 16-SWA-008_S64_R1.fastq.gz | G | - | - | - | - | - | - | - | - | T | - | - | - | T | - | - | - | - |
| 16-SWA-N058_S62_R1.fastq.gz | G | - | - | - | - | - | - | - | - | T | - | - | - | T | - | - | - | - |
| 16-SWA-0024_S53_R1.fastq.gz | G | - | - | - | - | - | - | - | - | T | - | - | - | T | - | - | - | - |
| 17-SWA-Q031_S85_R1.fastq.gz | G | - | - | - | - | - | - | - | - | T | - | - | - | T | - | - | - | - |
| 16-SWA-0023_S42_R1.fastq.gz | G | - | - | - | - | - | - | - | - | T | - | - | - | T | - | - | - | - |
| 17-SWA-Q06_S33_R1.fastq.gz | G | - | - | - | - | - | - | - | - | T | - | - | - | T | - | - | - | - |
| 16-SWA-N060_S86_R1.fastq.gz | G | - | - | - | - | - | - | - | - | T | - | - | - | T | - | - | A | T |
| SRR5585070 | G | - | - | - | - | - | - | - | - | T | - | - | - | T | - | - | - | - |
| SRR3049386 | G | - | - | - | - | - | - | - | - | T | - | - | - | T | - | - | A | T |
| 17-SWA-Q12_S11_R1.fastq.gz | - | - | - | - | - | - | - | - | - | - | - | - | - | - | - | - | - | - |
| 16-SWA-012_S57_R1.fastq.gz | - | - | - | - | - | - | - | - | - | - | - | - | - | - | - | - | A | T |
| 16-SWA-0034_S65_R1.fastq.gz | - | - | - | - | - | - | - | - | - | - | - | - | C | - | - | - | - | - |
| 16-SWA-N057_S50_R1.fastq.gz | - | - | - | - | - | - | - | - | - | - | - | - | C | - | - | - | - | - |
| 16-SWA-0017_S31_R1.fastq.gz | - | - | - | - | - | - | - | - | - | - | - | - | C | - | - | - | - | - |
| 16-SWA-005_S52_R1.fastq.gz | - | - | - | - | - | - | - | - | - | - | - | - | C | - | - | - | - | - |
| 17-SWA-Q14_S34_R1.fastq.gz | - | - | - | - | - | - | - | - | - | - | - | - | C | - | - | - | - | - |
| 16-SWA-004_S42_R1.fastq.gz | - | - | - | - | - | - | - | - | - | - | - | - | - | - | - | - | - | - |
| 17-SWA-Q030_S73_R1.fastq.gz | - | - | T | - | - | - | - | - | - | - | - | - | - | - | - | - | A | T |
| 17-SWA-Q18_S78_R1.fastq.gz | - | - | T | - | - | - | - | - | - | - | - | - | - | - | - | - | - | - |
| 16-SWA-N46_S32_R1.fastq.gz | - | - | T | - | - | - | - | - | - | - | - | - | - | - | - | - | - | - |
| 16-SWA-N055_S26_R1.fastq.gz | - | - | T | - | - | - | - | - | - | - | - | - | - | - | - | - | - | - |
| 17-SWA-Q20_S1_R1.fastq.gz | - | - | T | - | - | - | - | - | - | - | - | - | - | - | - | - | - | - |
| 16-SWA-N45_S21_R1.fastq.gz | - | - | T | - | - | - | - | - | - | - | - | - | - | - | - | - | - | - |
| 17-SWA-Q03_S87_R1.fastq.gz | - | - | - | - | - | - | - | - | - | - | - | - | - | - | - | - | - | - |
| 17-SWA-Q05_S22_R1.fastq.gz | - | - | T | - | - | - | - | - | - | - | - | - | - | - | - | - | - | - |
| 16-SWA-007_S63_R1.fastq.gz | - | - | - | - | - | - | - | - | - | - | - | - | - | - | - | - | - | - |
| 16-SWA-0028_S84_R1.fastq.gz | - | - | - | - | - | - | - | - | - | - | - | - | - | - | - | - | - | - |
| 16-SWA-N43_S86_R1.fastq.gz | - | - | - | - | - | - | - | - | - | - | - | - | - | - | - | - | - | - |
| 16-SWA-006_S62_R1.fastq.gz | - | - | - | - | - | - | - | - | - | - | - | - | - | - | - | - | - | - |
| 16-SWA-0031_S32_R1.fastq.gz | - | - | - | - | - | - | - | - | - | - | - | - | - | - | - | - | - | - |
| 17-SWA-018_S31_R1.fastq.gz | - | - | - | - | - | - | - | - | - | - | - | - | - | - | - | - | A | T |
| 17-SWA-019_S41_R1.fastq.gz | - | - | - | - | - | - | - | - | - | - | - | - | - | - | - | - | - | - |
| 16-SWA-0029_S10_R1.fastq.gz | - | - | - | - | - | - | G | - | - | - | - | - | - | - | - | - | - | - |
| 16-SWA-N061_S3_R1.fastq.gz | - | - | - | - | - | - | G | - | - | - | - | - | - | - | - | - | - | - |
| 16-SWA-N062_S15_R1.fastq.gz | - | - | - | - | - | - | G | - | - | - | - | - | - | - | - | - | - | - |
| 16-SWA-0027_S74_R1.fastq.gz | - | A | - | - | - | - | G | - | - | - | - | - | - | - | - | - | - | - |
| 16-SWA-N063_S27_R1.fastq.gz | - | A | - | - | - | - | G | - | - | - | - | - | - | - | - | - | - | - |
| 16-SWA-N064_S39_R1.fastq.gz | - | A | - | - | - | - | G | - | - | - | - | - | - | - | - | - | - | - |
| 16-SWA-N41_S64_R1.fastq.gz | - | A | - | - | - | - | G | - | - | - | - | - | - | - | - | - | - | - |
| 16-SWA-N47_S43_R1.fastq.gz | - | A | - | - | - | - | G | - | - | - | - | - | - | - | - | - | - | - |
| 16-SWA-N49_S84_R1.fastq.gz | - | - | - | - | - | - | - | - | - | - | - | - | - | - | - | - | - | - |
| 16-SWA-003_S31_R1.fastq.gz | - | - | - | - | - | - | - | - | - | - | - | - | - | - | - | - | - | - |
| 16-SWA-002_S225_R1.fastq.gz | - | - | - | - | - | - | - | - | - | - | - | - | C | - | - | - | - | - |
| 16-SWA-N065_S51_R1.fastq.gz | - | - | - | - | - | - | - | - | - | - | - | - | C | - | - | - | - | - |
| 16-SWA-N066_S63_R1.fastq.gz | - | - | - | - | - | - | - | - | - | - | - | - | C | - | - | - | - | - |
| 17-SWA-Q27_S78_R1.fastq.gz | - | - | - | - | - | - | - | T | - | - | - | - | - | - | T | - | - | - |
| 17-SWA-Q29_S13_R1.fastq.gz | - | - | - | - | - | - | - | T | - | - | - | - | - | - | T | - | - | - |
| 17-SWA-Q28_S2_R1.fastq.gz | - | - | - | - | - | - | - | T | - | - | - | - | - | - | T | - | A | T |
| 17-SWA-Q23_S34_R1.fastq.gz | - | - | - | - | - | - | - | T | - | - | - | - | - | - | T | - | - | - |
| 16-SWA-0030_S21_R1.fastq.gz | - | - | - | A | - | - | - | - | T | - | A | A | - | - | - | - | - | - |
| 16-SWA-N37_S20_R1.fastq.gz | - | - | - | A | - | - | - | - | T | - | A | A | - | - | - | - | - | - |
| 16-SWA-0035_S82_R1.fastq.gz | - | - | - | A | - | - | - | - | T | - | A | A | - | - | - | - | A | T |
| 16-SWA-0016R_S201_R1 | - | - | - | A | - | - | - | - | T | - | A | A | - | - | - | - | - | - |
| 17-SWA-Q22_S23_R1.fastq.gz | - | - | - | A | - | - | - | - | T | - | A | A | - | - | - | - | - | - |
| 16-SWA-N38_S31_R1.fastq.gz | - | - | - | - | - | - | - | - | T | - | A | A | - | - | - | - | A | T |
| 17-SWA-Q24_S45_R1.fastq.gz | - | - | - | - | T | T | - | - | T | - | - | - | - | - | - | T | - | - |
| 17-SWA-Q17_S67_R1.fastq.gz | - | - | - | - | T | T | - | - | T | - | - | - | - | - | - | T | - | - |
| 17-SWA-025_S61_R1.fastq.gz | - | - | - | - | T | T | - | - | T | - | - | - | - | - | - | T | - | - |
| 16-SWA-001_S63_R1.fastq.gz | - | - | - | - | T | T | - | - | T | - | - | - | - | - | - | T | - | - |
| 17-SWA-Q04_S10_R1.fastq.gz | - | - | - | - | T | T | - | - | T | - | - | - | - | - | - | T | A | T |
| 16-SWA-010_S28_R1.fastq.gz | - | - | - | - | T | T | - | - | T | - | - | - | - | - | - | T | A | T |
| 16-SWA-0026_S64_R1.fastq.gz | - | - | - | - | T | T | - | - | T | - | - | - | - | - | - | T | - | - |
| 16-SWA-N50_S8_R1.fastq.gz | - | - | - | - | T | T | - | - | T | - | - | - | - | - | - | T | - | - |
| 17-SWA-Q10_S77_R1.fastq.gz | - | - | - | - | T | T | - | - | T | - | - | - | - | - | - | T | - | - |
| 16-SWA-014_S16_R1.fastq.gz | - | - | - | - | T | T | - | - | T | - | - | - | - | - | - | T | - | - |
| 16-SWA-N52_S30_R1.fastq.gz | - | - | - | - | T | T | - | - | T | - | - | - | - | - | - | T | - | - |
| 17-SWA-022_S51_R1.fastq.gz | - | - | - | - | T | T | - | - | T | - | - | - | - | - | - | T | - | - |
| 16-SWA-N39_S42_R1.fastq.gz | - | - | - | - | T | T | - | - | T | - | - | - | - | - | - | T | - | - |
| 16-SWA-N054_S14_R1.fastq.gz | - | - | - | - | T | T | - | - | T | - | - | - | - | - | - | T | - | - |
| 17-SWA-Q21_S12_R1.fastq.gz | - | - | - | - | T | T | - | - | T | - | - | - | - | - | - | T | - | - |
| 17-SWA-Q01_S65_R1.fastq.gz | - | - | - | - | T | T | - | - | T | - | - | - | - | - | - | T | - | - |
| 16-SWA-015_S23_R1.fastq.gz | - | - | - | - | - | - | - | - | - | - | - | - | - | - | - | - | A | T |
| 16-SWA-N44_S9_R1.fastq.gz | - | - | - | - | - | - | G | - | - | - | - | - | - | - | - | - | - | - |
| 16-SWA-N48_S54_R1.fastq.gz | - | - | - | - | - | - | - | - | - | - | - | - | - | - | - | - | A | T |
| 16-SWA-009_S18_R1.fastq.gz | - | - | - | - | - | - | - | - | - | - | - | - | - | - | - | - | - | - |
| 16-SWA-011_S38_R1.fastq.gz | G | - | - | - | - | - | - | - | - | T | - | - | - | T | - | - | - | - |
| SRR1646371 | - | A | - | - | - | - | G | - | - | - | - | - | - | - | - | - | - | - |
| SRR1959430 | - | A | - | - | - | - | G | - | - | - | - | - | - | - | - | A | - | - |
| SRR3322140 | - | A | - | - | - | - | G | - | - | - | - | - | - | - | - | A | - | - |
| SRR1967707 | - | A | - | - | - | - | G | - | - | - | - | - | - | - | - | - | - | - |
| SRR1967264 | - | A | - | - | - | - | G | - | - | - | - | - | - | - | - | - | - | - |
| SRR5632298 | - | A | - | - | - | - | G | - | - | - | - | - | - | - | - | - | - | - |

Table S4.2: Details of genes where SNPs were observed

| **Position** | **REF** | **ALT** | **Type** | **Ref AA** | **Alt AA** | **Gene** | **Product** |
| --- | --- | --- | --- | --- | --- | --- | --- |
| 248500 | T | G | NS | Q | P | yhhQ | Conserved hypothetical integral inner membrane protein. Uncharacterized ACR YhhQ family COG1738 |
| 283938 | G | A | NA* | NA* | NA* | gntK | gluconate kinase 1, Thermoresistant gluconokinase, gluconate kinase 1, Gluconate kinase, carbohydrate kinase, thermoresistant glucokinase family, Shikimate kinase |
| 433414 | C | T | NS | V | I | yedZ | Membrane protein YedZ, Flavocytochrome yedZ, putative sulfite oxidase subunit YedZ, Ferric reductase like transmembrane component |
| 786026 | G | A | NS | G | R | papD_1 | fimbrial chaperone protein StdC, Chaperone protein papD precursor, Gram-negative pili assembly chaperone, N-terminal domain |
| 1267327 | C | T | S | A | A | acrD | efflux pump, Acriflavine resistance protein B, Caminoglycoside/multidrug efflux system, RND transporter, hydrophobe/amphiphile efflux-1 (HAE1) family, AcrB/AcrD/AcrF family |
| 1277426 | C | T | NS | R | C | SBOV25411 | putative periplasmic protein, Protein of unknown function (DUF1176) |
| 1663573 | C | T | S | T | T | wcaC | Glycosyltransferase. putative glycosyl transferase, colanic acid biosynthesis glycosyltransferase WcaC |
| 1735968 | G | T | NS | R | S | yvqK | ATP/cobalamin adenosyltransferase, Cob(I)yrinic acid a, c-diamide adenosyltransferase, hypothetical protein, Uncharacterized conserved protein, ATP:cob(I)alamin adenosyltransferase |
| 1884609 | C | T | NS | Q | stop codon | SEESL791_  009475 | IrsA, Protein of unknown function (DUF1367) |
| 2034437 | G | A | S | E | E | ydiI | ydiI hotdog fold superfamily, Esterase YdiI, acyl-CoA esterase, Uncharacterized protein, possibly involved in aromatic compounds catabolism, uncharacterized domain 1, Thioesterase superfamily |
| 2447181 | G | A | NS | A | T | hns | product=DNA-binding protein h-ns, hypothetical protein, global DNA-binding transcriptional dual regulator H-NS, H-NS histone family |
| 3086281 | A | C | NS | N | T | kdpD | sensor protein KdpD, Sensor protein KdpD, sensor protein KdpD, Signal transduction histidine kinase regulating C4-dicarboxylate transport system, phosphate regulon sensor kinase PhoR, Osmosensitive K+ channel His kinase sensor domain |
| 3167398 | C | T | NS | A | V | citC | citrate lyase synthetase, [Citrate [pro-3S]-lyase] ligase, [citrate (pro-3S)-lyase] ligase, Citrate lyase ligase C-terminal domain |
| 3519795 | C | T | NS | G | D | wapA_6 | type IV secretion protein Rhs, Cell wall-associated polypeptide CWBP200, Uncharacterized conserved protein, YD repeat (two copies), RHS Repeat |
| 3560772 | G | T | S | V | V | yaeE | ABC transporter permease, D-methionine transport system permease protein metI, DL-methionine transporter permease subunit, ABC-type phosphate/phosphonate transport system, permease component, phosphonate ABC transporter, permease protein PhnE, Binding-protein-dependent transport system inner membrane component |
| 3666065 | C | A | NS | L | I | aceF_2 | dihydrolipoamide acetyltransferase, Dihydrolipoyllysine-residue acetyltransferase component of pyruvate dehydrogenase complex, pyruvate dehydrogenase dihydrolipoyltransacetylase Acetyl/propionyl-CoA carboxylase, alpha subunit, dihydrolipoyllysine-residue acetyltransferase, Biotin-requiring enzyme |
| 3666067 | G | T |  |  |  |  |  |

* No Assembly with SNP available. NS = Non-synonymous, S = Synonymous
