# Supplementary figures and images for: Genomic characterisation of Salmonella enterica serovar Wangata isolates obtained from different sources reveals low genomic diversity
Source: PLoS One. 2020 Feb 28;15(2):e0229697. doi: 10.1371/journal.pone.0229697 (PMC7048276; doi:10.1371/journal.pone.0229697)

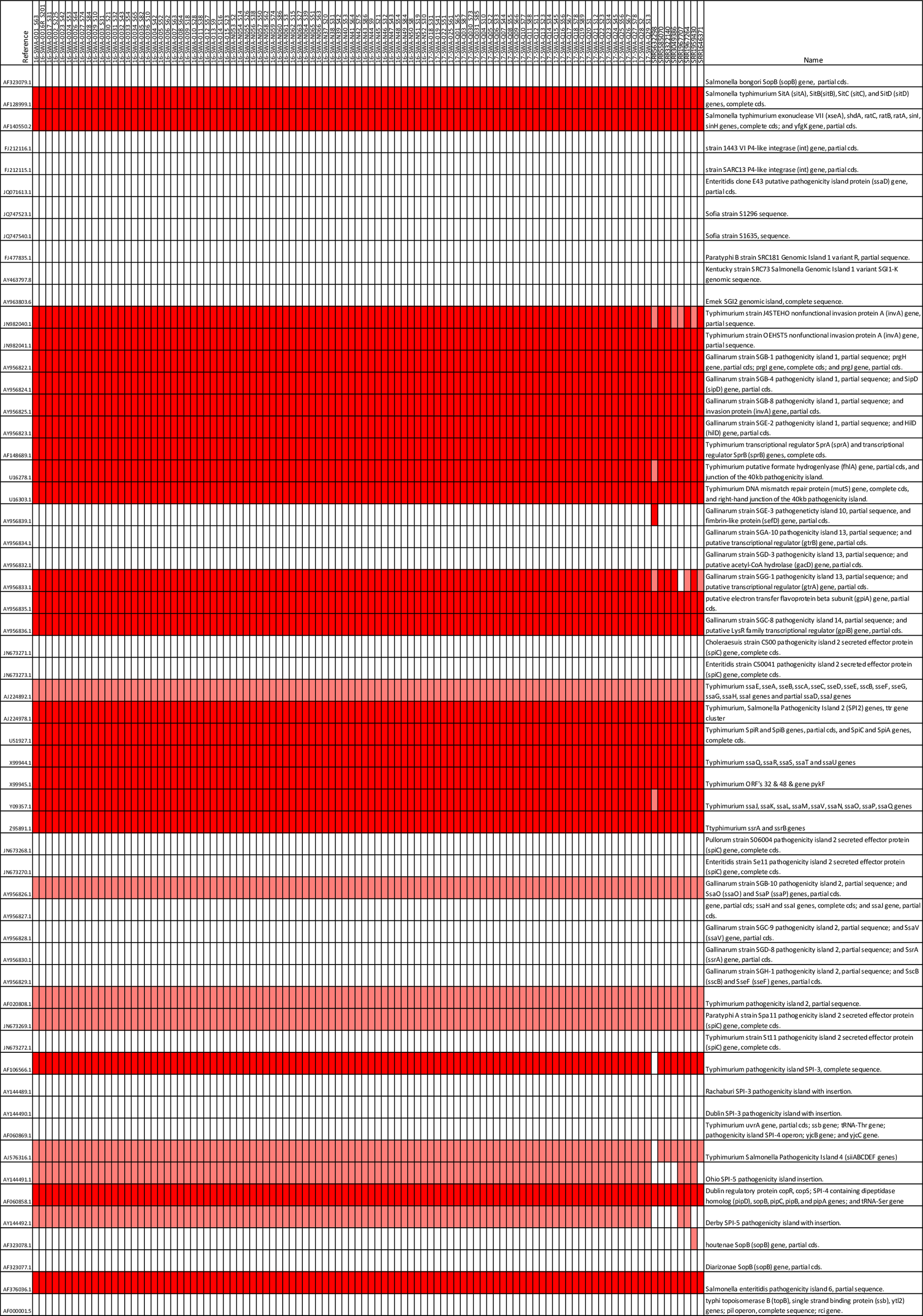

Supplement: S3 Fig — Pathogenicity islands were extracted from the Pathogenicity Island Database PAIDB 2.0 (www.paidb.re.kr/). Regions are defined as present (red), absent (white) or incomplete (pink) as determined by visual inspection of the coverage of reads mapped to the region. (TIF) [file pone.0229697.s005.tif]
